# Applications of Online UV-Vis Spectrophotometer for Drinking Water Quality Monitoring and Process Control: A Review

**DOI:** 10.3390/s22082987

**Published:** 2022-04-13

**Authors:** Zhining Shi, Christopher W. K. Chow, Rolando Fabris, Jixue Liu, Bo Jin

**Affiliations:** 1 School of Chemical Engineering and Advanced Materials, The University of Adelaide, Adelaide, SA 5005, Australia; zhining.shi@adelaide.edu.au (Z.S.); bo.jin@adelaide.edu.au (B.J.); 2 Sustainable Infrastructure and Resource Management, UniSA STEM, University of South Australia, Mawson Lakes, SA 5095, Australia; 3 Future Industries Institute, University of South Australia, Mawson Lakes, SA 5095, Australia; 4 South Australia Water Corporation, Adelaide, SA 5000, Australia; rolando.fabris@sawater.com.au; 5 UniSA STEM, University of South Australia, Mawson Lakes, SA 5095, Australia; jixue.liu@unisa.edu.au

**Keywords:** online UV-Vis spectrophotometer, real-time measurement, online water quality monitoring, drinking water

## Abstract

Water quality monitoring is an essential component of water quality management for water utilities for managing the drinking water supply. Online UV-Vis spectrophotometers are becoming popular choices for online water quality monitoring and process control, as they are reagent free, do not require sample pre-treatments and can provide continuous measurements. The advantages of the online UV-Vis sensors are that they can capture events and allow quicker responses to water quality changes compared to conventional water quality monitoring. This review summarizes the applications of online UV-Vis spectrophotometers for drinking water quality management in the last two decades. Water quality measurements can be performed directly using the built-in generic algorithms of the online UV-Vis instruments, including absorbance at 254 nm (UV_254_), colour, dissolved organic carbon (DOC), total organic carbon (TOC), turbidity and nitrate. To enhance the usability of this technique by providing a higher level of operations intelligence, the UV-Vis spectra combined with chemometrics approach offers simplicity, flexibility and applicability. The use of anomaly detection and an early warning was also discussed for drinking water quality monitoring at the source or in the distribution system. As most of the online UV-Vis instruments studies in the drinking water field were conducted at the laboratory- and pilot-scale, future work is needed for industrial-scale evaluation with ab appropriate validation methodology. Issues and potential solutions associated with online instruments for water quality monitoring have been provided. Current technique development outcomes indicate that future research and development work is needed for the integration of early warnings and real-time water treatment process control systems using the online UV-Vis spectrophotometers as part of the water quality management system.

## 1. Introduction

Drinking water quality is a key performance indicator for water utilities, and it is important for public health. Water utilities are committed to drinking-water quality management to ensure that the supplied water meets the drinking-water standards. Water quality management systems have been developed by water utilities to manage the water supply from catchment to tap, covering source water, water treatment and the distribution system for the safe supply of drinking water. Most drinking water quality management systems have followed the World Health Organization drinking water quality guidelines, with individual countries developing their own to manage their local situations. Water quality monitoring and treatment are two vital aspects of water quality management systems to detect hazards and events that can compromise water quality and provide operational control for assuring safe and reliable drinking water as preventive measures.

Water quality monitoring is needed to ensure the supplied water to the consumers meets the standards. Conventionally, water quality monitoring for drinking-water treatment relies on a regular sampling program (collection and transportation, followed by laboratory analysis), which often only captures small snapshots over a period of time and may not represent the true variances of water quality. It also frequently suffers from feedback delay and is unable to provide a rapid response to water incidents [1], as water analysis using standard laboratory methods often requires a longer processing time, such as sample pre-treatment or adding reagents. There is also a higher risk that the conventional monitoring method may miss water events that could lead to negative impacts on water quality and treatment process management. In contrast, online monitoring measures water quality continuously, which allows real-time water quality measurements and process control [2]. In summary, online water quality monitoring can improve the treatment process with the real-time assessment of both source and treated water quality, identification of contaminants and control of treatment process [3]. It is also useful during the period of rapid water quality changes when quick responses are needed to optimise the process [4].

There are many well-established online water quality sensors, including chlorine, total organic carbon (TOC), turbidity, conductivity, temperature and pH sensors, which normally only measure one water quality parameter. Two main types of sensors, biosensors and optical sensors, have received a lot of attention in recent years for online water quality monitoring [1,5]. Biosensors are mainly based on fluorescence and are used for the detection of microorganisms such as bacteria and viruses. Optical sensors measure light absorption, light scattering or fluorescence. A classic example of an optical sensor is a turbidity meter. More advanced optical sensors are infrared, fluorescence and UV-Vis spectrophotometers. Infrared optical sensors can continuously measure organic compounds at wavelengths greater than 760 nm and can analyse water samples at liquid or gas phases [6]. The infrared sensors are not commonly used for online water quality for water treatment. Florescence sensors can continuously determine dissolved organic matter and indicators of the microbial quality of water by analysing fluorescence from a molecule according to its fluorescent properties. UV-Vis sensors can also continuously measure water quality parameters by determining the amount of light absorbed by compounds, such as TOC and dissolved organic carbon (DOC), colour, nitrate and specialist parameters. Both florescence and UV-Vis sensors do not require sample pre-treatment, are reagent free and allow fast measurements [5], but UV-Vis sensors can measure multiple parameters for water quality monitoring and treatment process control.

Water quality analysis using UV-Vis spectrophotometers is a simple but effective method to provide measurements of water quality parameters. For conventional laboratory-based water quality monitoring procedures using spectrophotometers, sample pre-treatment is needed, such as physical filtration using 0.45 μm filters to eliminate particle interference for measuring UV_254_ and reagents for nitrate determinations. However, some commercial online UV-Vis spectrophotometers have built-in particle compensation and other algorithms [7] to eliminate sample pre-treatment and can provide calculated equivalents of water quality parameters such as UV_254_, colour, nitrate, DOC and TOC.

In recent years, additional parameters have been included in water quality monitoring using online UV-Vis spectrophotometers [8], such as measurements of dissolved organic matter [9], chemical oxygen demand (COD) in water bodies [10] and disinfectant in drinking water [11]. It has gradually been applied for the process control of water treatment, particularly for the coagulation process [12]. However, it can also be challenging to obtain accurate water quality measurements for those online UV-Vis instruments with built-in algorithms, with issues including under-compensation, over-compensation and failure to generate reasonable measurements for real-time monitoring and process control [13,14,15,16]. In contrast, reliable measurements were also reported using the online UV-Vis instruments for water quality detection and water treatment process control [17,18,19]. Various studies have been conducted to develop algorithms based on certain wavelengths (regions) of UV-Vis spectra to determine water quality, such as the use of absorbance ratios to monitor the variation of DOC in the water and multiple linear regression to estimate the total carbon contents in water [20,21].

Applications of UV-Vis spectrophotometers for water quality analysis have been reported in several review articles. A brief product review on a submersible UV-Vis spectrophotometer (probe) was conducted in 2006 which summarised the typical applications for wastewater treatment, environmental monitoring and drinking water applications [22]. The use of UV-Vis spectrophotometers for dissolved organic matter studies was reviewed, which summarised the use of derivatives and differential absorption spectra methods for DOC determinations in 2017 [9]. A recent review has been conducted on the advances of water quality detection by UV-Vis spectrophotometers in 2020 [8]. In the review, the principle of the instruments and modelling methods of predicting water quality were outlined. These reviews validate the principles of UV-Vis spectrophotometers and the general use of UV-Vis spectrophotometers.

As mentioned above, there were only several published reviews on the industry application of UV-Vis spectrophotometers. These reviews either were presented in a broad view, concluded the principles of the instruments or focused on a particular water quality parameter. Due to the lack of published research covering the practical aspect of using online UV-Vis spectrophotometers in drinking water supply applications, there is a need to expand the literature search to cover not only journal articles and books, but also existing guidance documents and industry reports in the applications of online UV-Vis instruments and identify the knowledge gaps [16,23]. Therefore, this paper presents an overview of the status and research progress of the UV-Vis instruments for online water quality monitoring and process control, particularly for industrial applications and the practical knowledge that makes UV-Vis instruments more acceptable to drinking water utilities. Firstly, an overview of online UV-Vis instrumentation is presented, followed by a brief discussion of online water quality monitoring using UV-Vis spectrophotometers for anomaly detection and early warnings. Finally, field applications of online UV-Vis spectrophotometers and their integration into the water quality management system is discussed. Challenges and solutions associated with the development and application of the online UV-Vis spectrophotometers for water quality monitoring are addressed. This paper also provides the researchers’ view for future research needs in the development and applications of online UV-Vis spectrophotometers.

## 2. Online UV-Vis Spectrophotometers

Online UV-Vis spectrophotometers can be effective and practical for measuring water quality parameters continuously and without the need for physical filtration using software particle compensation techniques. The water industry has deployed more online instruments to monitor water quality from catchment to tap for online and in-situ measurements as well as the treatment process control. However, the reputation of lacking reliability of the measurements is the general restriction of these instruments to expand to a wider range of water quality management applications. This section discusses those issues and limitations.

### 2.1. Online UV-Vis Spectroscopic Instrumentation

With the advancement of photodetectors’ development, there is an increasing variety of online UV-Vis spectrophotometers. Various UV-Vis sensors developed from different detection technologies and instrument designs are available for water quality monitoring and process control. The principle of UV-Vis spectrophotometry is based on the substance molecules in the water that can absorb UV-Vis light of a specific wavelength and the correlation between absorption spectrum and the concentration of the substance [8]. These sensing devices generally do not require sample filtrations (software particle compensation), are reagent free and allow fast measurements of water quality. They also have low maintenance requirements for parameters such as UV_254_ and the spectral absorption coefficient (SAC254); in addition, they are equipped with automatic ultrasonic cleaning systems without the need for manual cleaning; however, in some situations, particularly for turbid source waters, regular/on-demand manual cleanings of the measurement ports are still required to ensure reliable measurements. Commonly used commercially available online UV-Vis instruments are summarised in Table 1. There are mainly two categories of these online instruments: single wavelength (SW) and spectrum (full or partial).

Online SW UV-Vis instruments determine concentrations of a particular parameter in water based on the absorbance of a selected single wavelength [24]. The SW UV-Vis instruments, also called UV sensors, are usually manufactured with a specific wavelength to measure UV_254_ or nitrogen as nitrate and nitrite. The most common SW instruments are UV_254_ sensors which measure the absorbance at 254 nm with the absorbance at 550 nm for particle compensation. UV_254_ sensors can generate a surrogate parameter—SAC254 to determine dissolved organics and provide measurements of correlated parameters such as DOC and COD [24]. These surrogate parameters determined by the sensors are generated based on the correlations (often linear) of UV_254_ and the parameters, which is the wavelength of 254 nm absorbed by organic matter in the water. This concept is used by some commercial instruments including the HACH UV probe [25], Burkert SAC254 sensor [26] and YSI UV-Vis sensor; they use a single wavelength (absorbance at 550 nm) to compensate for the particle effect. These instruments are often SW instruments that use absorbance at 254 nm to determine the concentrations of a particular parameter, such as DOC.

In comparison to the SW UV sensors, UV-Vis spectral or full-spectrum sensors record the absorbances of a certain band of wavelengths or full spectra. These sensors produce fingerprints of spectra which are then used to determine and calculate concentrations of water quality parameters based on the instrument’s built-in algorithms. The spectral sensors can provide measurements for various parameters such as UV_254_, colour, DOC and turbidity using the algorithms. These instruments are generally factory calibrated for the particular water quality parameters using their proprietary algorithms but with site-specific re-calibration options. The instrument built-in proprietary algorithms are first used to remove the particle’s effect on the measurements of the water to replace the physical filtration step [27]. Some instruments, such as the IQ Sensor NET, provide surrogate parameters for DOC and COD, but the results are often not comparable to standard laboratory methods without specific calibration. This is because the correlations between the surrogate and standard analytical methods depend on the compositions of the water [18]. Re-calibration is often needed if significant changes happen in the compositions of water [18,28], which is different to the specific water type used in the original algorithm development [27].

Generally, when comparing the performance of full-spectrum and SW sensors, SW sensors can provide measurements and trends of the parameters varied during certain periods but may not compensate for the particle effect accurately, particularly when comparing the results with the standard laboratory procedures and measurements. The SW sensors may only provide a rough surrogate measurement of organic content and the total nitrogen content of nitrate and nitrite, and they do not have accurate particle compensation for most surface waters. In comparison, the spectral sensors provide better particle compensation and can be calibrated to specific locations with higher accuracy; they are better for precise applications, such as real-time water monitoring and treatment processes. In addition, the calibrations of online sensors are normally based on the grab sample collected at the same time of the measurement compared against laboratory measurement of that same water sample; these calibration procedures are more susceptible to the errors of grab samples.

**Table 1:** Summary of common online UV-Vis instruments for water quality monitoring and process control.

Sensor	Manufacturer	Optical System	Measured Wavelength	Measured Parameter	Advantages	Accuracy	Operating Range	Source
AMI SAC254	SWAN, Switzerland	Two-wavelength photometer with one optical channel, light-emitting diode (LED) light	254 nm	Surrogate parameter to determine dissolved organics	Measuring interval: 30 s to 3 min	±1% m^−1^	UV absorption: 0–6 mg/L DOC, TOC: 0–6 mg/L SAC254: 0 to 300 m^−1^ Temperature: 5–30 °C	[29]
ProPS-UV Photometer	Trios GmbH, Germany	Detector type: UV spectrometer, light source: deuterium lamp	200–385 nm	nitrate, CODeq and TOCeq	Customize path lengths, Spectral analysing software, Additional calibration functions	±0.01% mg/L	Temperature 0–30 °C, 32–86 °C; Measurements: 0.62–600 mg/L	[30]
IQ Sensor NET	WTW GmbH, Germany	256 channel silicon photodiode array detector, deuterium lamp	200–720 nm	A range of parameters, e.g., SAC, UVT	Data logger	±3% mg/L	SAC: 0.0–3000 m^−1^ Temperature: 0–45 °C	[31]
spectro::lyser	s::can Messtechnik GmbH, Austria	256-pixel photodiode array detector, xenon flash lamp	200–720 nm 220–390 nm	Various parameters	Various parametersDiffer path lengths	±2% mg/L	Temperature: 0–45 °C; TOC: 0–180 mg/L; NO_2_-N: 0–40 mg/L; NO_3_-N: 0–100 mg/L; UV_254_: 0–500 abs/m	[32]
Real UV254 probe	RealTech, Germany	Mercury UV lamp and LED lamp	253.7 nm	SAC254	Various parametersVarious path lengthsField calibration	±5% m^−1^	Temperature: 0 to 45 °C UV254: 0–20 abs/cm	[33]
UV absorption sensor	Endress+Hauser, Switzerland	Hotovoltatic cells detector, low-pressure mercury lamp	254 nm	SAC254	Data logger	±3% m^−1^	0–2.5 abs/cm 0 to 90 °C	[34]
IQ SensorNet system	YSI, Germany	Detector: LED and photodiode	254 nm	UVT-254 and SAC254	Has a controller	±2% m^−1^	Temperature 0 to 45 °C; UVT-254: 0–100; SAC254 0–3000 m^−1^	[35]

### 2.2. Water Quality Measurements with Proprietary Algorithms

As reported earlier, some advanced full-spectrum online UV-Vis spectrophotometers can determine a range of water quality parameters, including UV_254_, colour, DOC, turbidity and nitrate. The parameters can be computed based on the applications via the instrument’s built-in proprietary algorithms. According to the manufacturer literature, these algorithms were developed based on chemometrics techniques, such as partial least squares (PLS) and multiple linear regression, to establish the relationship between UV-Vis spectra and laboratory measurements of water samples [7]. The algorithms were created by hundreds of global datasets containing both UV-Vis spectra and reference laboratory data obtained from a wide range of water quality [7]. Instruments have default configurations to apply the generic calibration for particle compensation to the raw spectral data using the built-in algorithms.

UV_254_ measures the amount of light absorbed by conjugated organic compounds, which have been widely used as a rapid water quality measurement technique to control water treatment processes [36]. UV_254_ from the online UV-Vis instruments based on built-in algorithms generally performs well for the treated water as less interference exists [27]. However, performing site-specific calibrations may be needed if it is used for source water with a complex matrix. It is more difficult to judge the accuracy of the colour measurement than UV_254_ using the online UV-Vis instruments, as the standard laboratory colour measurement method relies on SW measurement. Besides, the wavelength selected to measure colour may be different based on regions and water sources; for instance, 456 nm is used by Australia and the USA, whereas 410 nm is used by Russia to measure colour in natural water [37]. To measure the colour of water, the online instrument needs to be set up according to the required wavelength. DOC is used to monitor water quality from catchment to tap water, and measurement is usually carried out using the laboratory-based standard method. Online UV-Vis instruments with built-in algorithms can be used as alternative measurements; however, they are often reported as water-specific and lacking accuracy and thus need additional calibrations against different water matrices [38]. Turbidity determined by the online UV-Vis spectrophotometers with the generic built-in algorithms is comparable to the turbidity results analysed in the laboratory [39,40]. Nitrates generated from the online instruments with the generic built-in algorithms are generally not satisfactory [39], but another group reported that the results are comparable with a laboratory analysis [40]; this could be related to the specific algorithm used and the monitoring application.

The measurements of water quality parameters using the online UV-Vis spectrophotometers are often source-water dependent. Thus, additional site-specific calibrations are needed to improve the accuracy of measurements [7,18,41,42,43] which is an instrument function provided to enter the laboratory-determined value of the collected grab sample measured at the same time by the online instrument. The site-specific calibration process involves modifying the slope and intercept of the built-in regression function using laboratory data from the reference grab samples [18,41]. To achieve the best calibration results, grab samples are needed to be representative and cover the whole measurement range of the water. Measurement accuracies can be enhanced with the use of an increased number of grab samples for the calibrations of the online UV-Vis spectrophotometers.

Applications of online UV-Vis instruments can reveal that the significant fluctuations of water quality could affect the accuracy of the measurements, and long-term monitoring required regular calibration to compensate for the variation of the particle character issue [16,27,41]. A site-specific calibration was conducted for a submersible UV-Vis instrument to monitor water quality in a forested catchment, and comparable results were achieved [42]. In contrast, a site-specific calibration was performed for a UV-Vis submersible instrument to measure the water quality of stream water, but concentrations were overestimated because of inaccurate particle compensation [43]. Therefore, accurate site-specific calibration of the UV-Vis instrument is crucial to obtain measurements for water quality monitoring.

Calibrations of online UV-Vis instruments should be performed as needed for situations such as an instrument being installed for the first time, changing the monitoring locations of the instrument, or the instrument being inaccurate to the reference water samples. Routine calibration may not be necessary for monitoring less variable source water or stable water quality, such as treated water, but routine verification of the measurements using lab references is recommended to ensure the accuracies of the instruments [38]. Site-specific calibrations have been approved to achieve the desirable measurement outcomes and can adequately account for the differences in large water quality changes or between different types of water [13,15,44,45,46].

### 2.3. User-Developed Algorithms for Spectral Absorbance Measurements

When there are difficulties to obtain accurate continuous measurements using online UV-Vis spectrophotometers with the instrument built-in algorithms or when the instruments do not come with built-in algorithms, alternative particle compensation (calibration) techniques can be developed by end-users. To better utilise the online instruments for water quality monitoring and water treatment process control, as well as lower the maintenance costs, more importantly gaining in-house experience and knowledge of the instruments, researchers and water utilities have been seeking techniques for particle compensation alternative to the built-in particle compensation methods. The particle compensation techniques based on the UV-Vis spectra can be categorised into direct subtraction compensation and chemometric modelling. Table 2 summarises particle compensation techniques from the literature for online water quality monitoring using the UV-Vis instruments.

**Figure 1:**
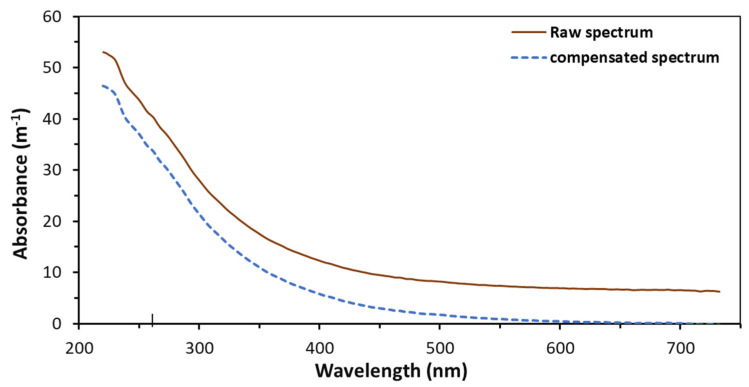
Illustration of particle compensation of a raw spectrum for surface water using a single-wavelength method.

Direct subtraction compensation is based on the absorbance of wavelength characterised by the particles in the water [7,59]. Wavelengths, including 275 nm, 350 nm, 545 nm, 546 nm and 550 nm, have been utilised to characterise the particles in the water and to remove the particle effect from the UV-Vis measurements [47,48,49]. The absorbance at 546 nm was used to remove the particle effect on the COD in river water [48]. The wavelength at 545 nm was used to reduce the particle influence on the UV for surface water [49]. The wavelength at 550 nm is commonly selected for SW particle compensation for individual water quality parameters and has often been used for the measurements of UV_254_ [27]. UV at 350 nm has been used to compensate for the online measurements of COD using UV spectrophotometry to detect groundwater quality to remove the influence from particles [47]. In the same study, the absorbance at 275 nm was also used to compensate for nitrate at 220 nm. Figure 1 shows an example of using an SW particle compensation method to remove the particle’s influence on a raw spectrum for surface water [27].

The multi-wavelength particle compensation technique, also called chemometric modelling, includes a selection of chemometrics such as multiple linear regression (MLR), multiple stepwise regression (MSR), support vector machine (SVM), support vector regression (SVR), multiplicative scatter correction (MSC), principal-component analysis, and PLS. The multi-wavelength particle compensation technique is based on relationships between the raw spectra and laboratory reference values of the water quality parameters. The MLR determines the linear relationship between a dependent variable (the laboratory values) and independent variables (wavelengths of the raw spectra) which can directly define the coefficient of each variable. MLR was employed to remove the particle effect on the UV-Vis spectra of brackish water for the rapid measurement of water quality parameters [56]. Multiple linear regression was also used to quantify the DOC content in the stream water [17] and TOC in the drinking water, seawater and river water [21]. Multiple stepwise regression was used to compensate for the particle effect on the DOC measurements for surface water [57]. SVM is a machine learning algorithm that can be used for classification, regression and outlier detection. SVM was used to determine the concentration of dissolved nutrients in surface water using the full spectral wavelengths and laboratory values and demonstrated the effectiveness of the approach [58]. SVR is similar machine learning method as SVM, but it works with continuous values instead of classification, as in SVM. SVR was used to predict the combined nitrate and nitrite concentration for treated water samples using spectral features, and the predicted values were matched with the standard laboratory values [60]. MSC is a normalization technique to correct the particle’s effect on spectra by changing the scale and the offset based on the reference spectrum, which is the mean of the spectra. The MSC method was applied to compensate for the particle’s effect on the COD in lake water [19], UV_254_ in reservoir water [27] simulated surface water [51] and DOC in drinking water production [52].

PLS constructs components by projecting the predictor variables to a new space. Then, the linear regression models were built between new predictors and responses. PLS can be used to extract important information from a large data matrix [61]. PLS regression is a commonly used method to remove the particle’s effect on the measurements of water quality parameters based on the multiple wavelength spectra. It has been used to remove the particle’s effect on the water quality multi-parameter, such as COD in artificial seawater [62], suspended solid in brackish water [56], COD in lake water [63], COD and TOC in seawater [54] and stream water [17], nitrate in water [53], nitrate and nitrite in seawater [64], DOC in surface water [57] and drinking water [52] and ozone in drinking water [55].

The use of a subtraction method for particle compensation generally works well for low- and medium-turbid source water and treated water, but it may lead to less accurate measurements in some cases, such as highly turbid water [50,57]. The accuracies of the measurements such as DOC in the surface waters can be improved by using the multi-wavelength particle compensation methods. Water matrix-specific particle compensation is often recommended for water quality monitoring. There are some benefits of using alternative particle compensation methods to the built-in algorithms. Firstly, it creates simplicity and flexibility of custom-made particle compensation methods for water quality measurements of the particulate water matrix, as the details of the built-in algorithms for the commercial online instruments are often not accessible to the users. Secondly, the use of alternative particle compensation methods can lower the calibration costs of the instruments. Moreover, cost-effective UV-Vis instruments with a single wavelength or short wavelength band could be employed in the field to monitor water quality instead of using full-spectrum UV-Vis instruments.

## 3. Advanced Spectral Data Processing and Applications

UV-Vis spectrophotometers can be used for real-time water quality monitoring and integrated with early warning systems to detect rapidly changing water quality. Water quality parameters, including turbidity, SAC254, nitrate, TOC and DOC, can be monitored and provide early warning. The warning occurs when the current measurements exceed limits that are specific to each parameter or anomalous patterns are detected [65], then the appropriate actions can be taken. One example is to monitor spring water; the spring water of concern would not be used for drinking water production when the measurements go beyond the limits of the measuring parameters [66]. The anomaly detection methods using the UV-Vis instruments can be easily configured for real-time monitoring of water pollution and early warnings [21,65,67].

UV-Vis spectra contain valuable information on the composition and quality of water and can be used as a fingerprint of the water matrix. The fingerprint can be used to derive specific parameters such as turbidity and DOC. Online UV-Vis instruments have a fingerprint, which can also be used to monitor changes in the water composition and offers the possibility to set alarm levels based on the magnitude of the variations in the spectra and early warning systems by water utilities [22,68]. It is an advanced spectral data processing system and can be applied for the early warning of anomaly detection and the identification of contaminants. Online UV-Vis instruments have been used by some water utilities to develop early warning systems to monitor drinking water quality at the source or in the distribution system for water quality control as a component of the drinking water quality management system. It can detect not only natural contamination, but also accidentally or intentional contamination.

### 3.1. Anomaly Detection of Water Quality

Quite often, water utilities face minor and major water quality incidents; the concerning major events can be extreme concentrations of water quality contents or pollutants or accidents of pollutant events. Rapid fluctuations of source water quality could happen. For instance, turbidity and organic matter could suddenly be raised by storm events. Quick detection of the water quality in response to the contaminant event is essential to reduce risks when water quality events occur. The use of online UV-Vis instruments allows near- or real-time detection of anomalies and contaminations of drinking water systems. Early detections are vital for effective responses that reduce or prevent contamination events that compromise water quality and avoid possible failures of WTP operation [68]. The use of water quality anomalies detection from the UV-Vis spectra contributes to the safety of water quality. Detection using the UV-Vis spectra for water quality monitoring is mostly applied for organic contaminant monitoring, as UV-Vis monitoring has the advantage reported earlier, without the need for sample preparation, being regent-free and having a low operational cost compared to the standard laboratory analysis of organics [21,69,70].

An anomaly detection method may have three components, including data analysis, event detection and performance assessment, which are able to provide a reliable indication of contamination by analysing the real-time water quality data [69]. The first step is to establish a baseline of the stable water quality in the normal condition. Data analysis is performed to remove the particle effect, instrument noise and drifting from the water quality measurements. Event detection is performed to analyse the real-time water quality data by comparing the pattern of new data with the pattern of normal data based on machine learning and chemometrics. Performance assessment is conducted to evaluate the detection method to meet the required accuracy [69].

A proximate entropy approach was applied to measure the UV-Vis spectra and differentiate the normal and abnormal spectra of water in distribution systems. This method had a good detention outcome [57]. In addition, the fitness measure which combined both the Pearson correlation and the Euclidean distance was assessed as a technique to identify contaminated water from drinking water using a submersible UV-Vis instrument in a controlled study [71]. The method was flexible to identify the source of water and distinguish the contaminated water. This method was further tested to cope with various backgrounds with changing proportions of water from different sources using a combination of UV-Vis spectral data from both laboratory experiments and an operational water supply system [67]. The detections were based on combinational changes of water sources and operational and maintenance actions. Contaminants at low concentrations were detected.

### 3.2. Early Warning Systems for Water Supply

An early warning system integrated with a UV-Vis spectrophotometer has been extensively tested at a laboratory-scale and has achieved robust results. The interesting aspect of the early warning system is that it not only detects and quantifies specific compounds, but also detects unknown compounds that do not fit in the normal fluctuation of the water matrix [72]. Alarm parameters can be developed from the spectral data, and abrupt spectral signals can be extracted by using anomaly detection techniques. The process of alarm development of water quality monitoring includes a learning period, abnormality definition, alarm level definition and sensitivity definition [73]. Various methods have been used to identify anomaly events, such as a probabilistic principal component analysis (PPCA), Bayesian algorithm, principal component analysis and Euclidean distance method [65,74,75]. A PPCA-based method was used to identify anomaly events with the employment of online UV-Vis instruments. The PPCA algorithm was used to simplify the large amount of spectra data and retain the essential spectral information. It was tested for online water quality monitoring in a small-scale water distribution system [75]. The PPCA method was combined with a multivariate monitoring chart to provide a reliable and flexible alarm system. The Bayesian algorithm combined with a UV-Vis spectrometry probe, along with a message-passing schedule, was used to analyse patterns for event classification. It was conducted for long-term online monitoring of the water distribution system in a pilot-scale [76]. Water quality anomalies were detected using the integration of the principal component analysis and chi-square distribution combined with UV-Vis sensors for a distribution system. It was conducted at a pilot-scale and proved to be a promising method [74]. In addition, an early warning system was used for remote river water quality monitoring for COD content and early detection, which had a real-time display and storage and warning functions [35].

Early warning systems need to be able to identify whether variation in sensor measurements is caused by equipment noise or the presence of contamination or high levels of concentrations. Methods including the Pearson correlation Euclidean distance-based method, multivariate Euclidean distance method and linear prediction filters method have been used to detect changes in water quality and differentiate between fluctuations caused by equipment noise and those due to contamination [77]. This method was able to detect 95% of contamination events correctly, with a 2% false alarm rate from a contaminant injection experiment [77]. The Pearson correlation Euclidean distance method was applied to a real contamination accident study; the results showed that this method has better potential to be used in the field [76].

Various water quality detection methods based on UV-Vis spectral data have been developed and assessed. However, the evaluation of detection performance is mainly based on a simulation or laboratory study. The reported evaluation of detection performance was rarely based on real contamination events. There are arguments that lab- and pilot-scaled studies may not cover the variation of water quality that occurs in the actual water systems, as real water quality data may contain more background noise and fluctuations [76]. Therefore, it is important to test the detection method in a real water event situation.

### 3.3. Integrated Early Warning and Real-Time Control System

**Figure 2:**
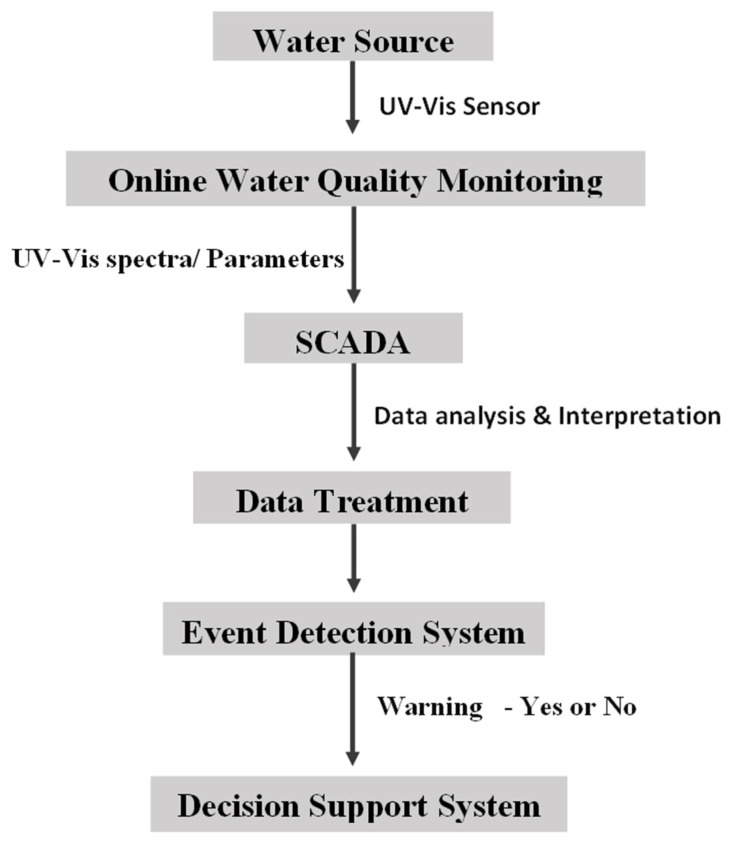
Structure of an early warning system.

An integrated early warning and real-time control system for drinking water combines the functions of water quality monitoring, an early warning system and decision making. It is an integrated approach to detect and respond to water quality events that use advanced monitoring technologies to provide warnings of potential contamination incidents and quick responses [75]. An integrated early warning system should contain an online monitoring system, supervisory control and data acquisition (SCADA) and event detection system and a decision support system as shown in Figure 2. Event detection provides indications of abnormal water conditions. Early warning systems should be able to quickly detect water quality and contamination events with high levels of accuracy, reliability, cost-effectiveness, being user-friendly and low maintenance [78]. This includes an integrated system with event-driven functions for detecting, reporting and handling water quality contamination events automatically in real-time.

An integrated system can provide water quality monitoring and warning performances to monitor hazard and forecast hazard evaluation and issue timely and accurate warnings of water quality anomalies. It fits well with the drinking water management system. Online UV-Vis instruments have been used as part of the integrated system to continuously provide water quality data. Studies have been conducted by deploying UV-Vis instruments for the real-time online analysis of water quality and anomaly detection, particularly in Europe and the United States [78]. A semi-supervised learning model combined with a UV-Vis spectra was used to detect organic contamination events successfully in water distribution systems. This adaptive method modified the baseline using a dynamic orthogonal projection correction and adjusts the support vector regression model in real-time [79]. A discrete wavelets transformation and a principal component analysis can also be used for detecting organic contamination events from UV-Vis spectral data. This approach was tested online using a pilot-scale setup and experimental data [65]. Abrupt changes of the spectra were captured, and an alarm of the contamination event was able to be identified.

Another event detection approach is based on UV-Vis signal processing and data-driven techniques. Early warning systems combine automatic measurements with automatic data evaluation and data transfer for water quality monitoring, such as surface water [80]. A web interface of the system works as a control centre constantly checking for anomalies in water quality based on automatic data evaluation. Maintenance can be reduced, as remote checking of water quality is available [81].

## 4. UV-Vis Spectrophotometer Application and Integration of the Water Quality Management System

The online UV-Vis spectrophotometers can continuously measure water quality online in real-time, and they have a broad application in drinking water networks through monitoring of source water quality, treatment processes and treated water as part of the drinking water quality management system. Many reported studies of the online instruments have been conducted at the laboratory-scale, as shown in Table 2. It has become widely recognised in the water industry that current applications of UV-Vis instruments in real-time for water quality monitoring remain limited. There is an increasing trend of using online UV-Vis instruments, especially in water quality monitoring and process control and as early warning systems [82]. The use of online UV-Vis instruments for water quality monitoring allows better water quality management compared to conventional water quality monitoring, as it supports continuous updating of water quality and can detect any potential water quality events and provides timely decision support. The ability of online UV-Vis instruments to detect issues in real-time to allow rapid response to any water quality event is valuable to the water quality management system [83]. It also allows for real-time understanding of operational causes, which, in turn, contributes to the optimisation of the water treatment processes.

### 4.1. Requirements and Supports of Using Online UV-Vis Spectrophotometers in Real Operations

**Figure 3:**
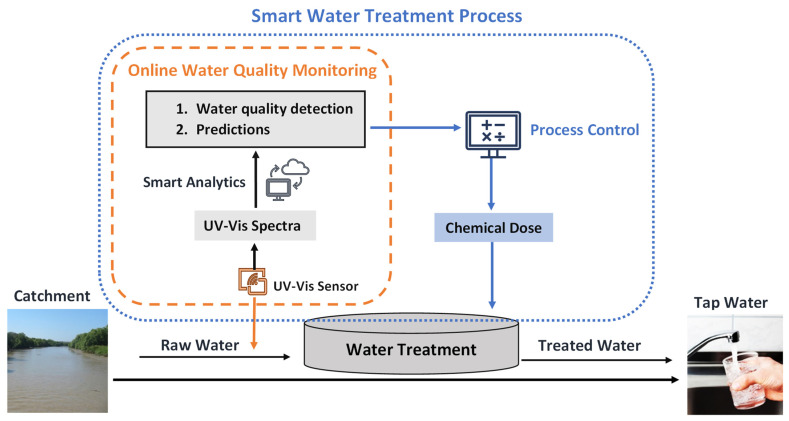
Applications of online UV-Vis sensors for real-time water quality monitoring and process control.

Online water quality monitoring using spectrophotometers allow a fast and effective response to water quality events. Online UV-Vis instruments have been used for determinations of process upset or deterioration in water quality, as well as operation and control of the drinking WTPs. Applications of the online UV-Vis instruments in water treatment and distribution networks can identify water quality parameters such as nitrate and organic pollutants rapidly and can measure and analyse the parameters simultaneously. An illustration of applications of online UV-Vis sensors for real-time water quality monitoring and process control is shown in Figure 3. Field applications of online UV-Vis spectrophotometers were summarised in Table 3. Most field applications of the online UV-Vis instruments were on water quality monitoring. Some case applications were conducted on anomaly detection and early warning systems. Very few cases were used for the process control of drinking WTP.

The most important applications of UV-Vis instruments are monitoring of the source water and treatment process control [22]. Field applications of UV-Vis instruments showed that the instruments are suitable for the estimation of DOC concentration. A study assessed the performance of a portable UV-Vis spectrometer in measuring the DOC concentrations of surface water under field conditions [57]. It demonstrated the possibility of facilitating rapid, robust and continuous measurements. A contaminant warning system was developed in Dallas, Texas, USA, to monitor the drinking water quality in the distribution system [84]. This warning system consisted of online UV-Vis instruments which provided a continuous analysis at 16 checkpoints in the distribution system. Anomalies were constantly checked for water quality parameters such as nitrate, total chlorine turbidity, TOC, conductivity, UV_254_, DOC, pH and free ammonia. All of the information is web-accessible to the operators for the detection of water quality changes at treatment plants.

The operation of drinking water treatment plants is mainly based on laboratory analyses of grab samples and the experiences of operators. In recent years, there has been an increasing need of using model-based monitoring for the optimisation and control of water treatment plants. However, most studies were conducted at a laboratory- or pilot-scale. The use of model-based monitoring has shifted the operation of drinking WTPs from experience-driven to knowledge-based [28]. Modelling, in combination with online monitoring and real-time control, can improve the treatment operation, leading to better control of a more stable water quality [85]. An online UV-Vis instrument was used for a feed-forward coagulant dose prediction to avoid the increase in turbidity of settled water and support the operation of a WTP. The predicted coagulant doses were used as inputs of the plant control system to automatically control the coagulant dose in response to the online measurements of raw water quality [86].

The online UV-Vis instruments combined with advanced data analysis techniques, such as machine learning, allow real-time water quality monitoring and provide valuable tools for effective water quality management. The combination of real-time water quality data and advanced data analysis techniques can be efficient for the management of water quality. The recent advances in technologies enable the application of web-based data platforms for analysing real-time data for water quality management. Efficient and real-time monitoring of water quality as a key component of water quality management can predict future trends of water quality and enable rapid response to water quality events [83].

**Table 3:** Summary of field applications of online UV-Vis spectrophotometers.

Water Type	Application	Measurement	Location	Reference
Surface water	Rea-time Monitor test filters	DOC, TOC	Danuba Island, Austria	[87]
River water	Real-time Monitor water quality	Nitrate, DOC, TSS	Kervidy-Naizi, West France	[88]
Stream water	In-situ Monitor stream DOC	DOC	South Korea	[89]
Drinking water	Online monitoring and process control	Surrogate A_254_, A_202_, A_290_, A_310_, A_350_,	SA Water, South Australia	[90]
Drinking water	Early warning system in the drinking water supply	Nitrate, TOC, SAC254	Bratislava Water Company Austria	[91]
Drinking water	Coagulant control	Turbidity, alum dose	Morgan WTP, South Australia	[86]
Drinking water	Measure dissolved ozone and AOC concentrations	Assailable organic carbon	Vienna Waterworks, Austria	[55]
Filtered water	Real-time Monitor water quality	UV254	SA Water, South Australia	[13]
Lake water	Monitor variation of carbon content	DOC using Absorbance at 285 nm	Lake Ipê, MS, Brazil	[20]
Surface water	Measure DOC content in situ	DOC	Europe	[18]
River water	Monitor water quality in situ	DOC, Fe	Krycklan river, Sweden	[17]
Surface water	Monitor dissolved nutrients in real-time	Nitrate	Windsor, Canada	[58]
Groundwater	High-resolution monitoring	Nitrate	Southwest Ireland	[41]
Stream water	Monitor storm events	DOC	Haean Basin, South Korea	[43]
River water	Real-time Monitor of water quality	NO3-N, DOC	Saarland	[46]
Spring water	Online monitoring	SAC254, Nitrate, TOC, DOC	Vienna Waterworks, Austria	[66]
Treated water	In situ anomaly detection	Spectra	Hangzhou, China	[92]
Drinking water	Online monitoring anomaly in water distribution systems	Spectra	Hangzhou, China	[75]
River water	Real-time monitoring	COD	Jialing River, China	[35]
Groundwater	Early warning	Nitrate, nitrite	Vienna, Austria	[80]
Drinking water	Contamination warning system	Spectra	Dallas, US	[84]
Fresh water	Simultaneous determination of nitrate and nitrite	nitrate and nitrite	UK	[30]
Spring water	Online Water-Quality Monitoring	SAC254	NW Switzerland	[34]

### 4.2. Challenges and Solutions of Using Online UV-Vis Spectrophotometers

Although many studies have shown that online UV-Vis spectroscopy can detect water quality changes, such as rapid detection of changes in the raw water quality, and allow for real-time adjustment of the process, there are still challenges remaining for the practical applications. There is also a lack of harmonisation of standards and regulatory practices of using online instruments for water quality monitoring [16]. Some regulatory guidelines of drinking water only mention online instruments in general terms but highlight the need for accurate measurements and recommend online continuous monitoring of water where possible. One of the main issues is the detection limit, as the field environment is complex. Another issue is the difficulty of detecting the UV-Vis spectra of some pollutants in the water, such as suspended solids, dissolved inorganic substances and pathogenic microorganisms. Most of the difficulties in using the online instrument are caused by the highly challenging nature of the source water. Solutions were developed to allow realisable monitoring of the source water, including determining appropriate manual cleaning intervals.

UV-Vis instruments generally work well for real-time monitoring for treated drinking water, as fewer interferences exist [58]. However, they have experienced measurement issues in field applications for source water quality monitoring, particularly for surface water that has complex chemical compositions. Field experience shows that the path lengths of the UV-Vis instruments had a significant influence on the sensitivity and the range of water quality parameters [41]. The selection of path length is related to the water matrix. The sensitivity increases with the path length. A longer path length leads to a higher sensitivity but a reduced maximum concentration level at which the instrument can operate [82]. The typical path length is within 0.5–100 mm. Normally, a path length of 100 mm is suitable for drinking water, 35 mm and 10 mm for surface water and 5 mm for wastewater applications. The natural variation occurrence can be determined, which requires measurements of the fingerprint spectrum across several months for training and local calibration of the instruments.

In the absence of site-specific calibration, the determination of DOC concentration can be inaccurate due to varying absorbance strengths of the interference of other elements in the water [57]. For instance, absorbance measurements can be influenced by changing of the source water [27]. The accuracy of the water quality measurements can be affected if the content, such as turbidity or organic matter, varies after the instrument has been calibrated to a particular water matrix [41]. Corresponding laboratory data should cover the seasonal variations of the site-specific water source for calibrating the instruments. Regular validations of the online measurements are needed to eliminate temporal drifts and maintain accuracy. The re-calibration of particle compensation could be complicated if the water matrix varies significantly and lacks support from experts.

Water operators may face various challenges in the use of the online UV-Vis instrument, including instrument maintenance, installation, data processing and variability in the parameter performance. Typical issues and solutions associated with the online UV-Vis spectrophotometers are summarised in Table 4. An example of installation issues of the online instrument is the accelerated probe corrosion issue caused by the jetty cathodic protection system in monitoring river water [82]. The solution to this problem was to use an ‘on-demand’ pump sampling system to protect the instrument from corrosion, reduce fouling by silt and biofilm and reduce the maintenance requirements.

Potential data storage and processing could be problematic in the online monitoring using the UV-Vis instruments. Online instruments can collect and store some acquired data but are not able to collate the data for easy access and interpretation. Pre-processing of the UV-Vis spectra data is required to assure the data quality, including the removal of faulty spectra and outliers, as well as the performance of particle compensation. The issue of data processing is that standard data storage and analysis programs such as Microsoft Excel cannot handle the large volume and high dimensional complexity of the UV-Vis spectra data. Development has been made in data processing with specialised tools to tackle the challenges [78]. An example of the specialised software, Visual Basic 6.0, has functions such as a selective display of water quality parameters, automatic detection of invalid data, automatic deletion of invalid data, the exportation of data from selective periods and also data resolution options, which allow easier plotting of long-term data [38]. Advanced systems need to be suitable for instrument operation in the long term. Expertise is needed to design and troubleshoot the program, and the data analytic system is needed to link with the control system, such as SCADA. An open-source Python toolbox called ‘AbspectroscoPY’ was developed for the pre-processing and analysis of the large volume of raw UV-Vis absorbance time-series data [3]. The toolbox has the functions of automated outlier detection and removal based on the interquartile range. Some online UV-Vis Spectrophotometers only provide water quality parameter data, in which case a simple data logger can be built to manage data collections. For example, a web data extraction was built with a Python library and data store to automatically monitor the water quality of reservoirs [31].

To achieve the best outcomes of water quality monitoring and process control using online UV-Vis sensors, the following operation steps are needed: (1) the instruments should be calibrated for new sites or source water changes over, (2) pre-treatment of the UV-Vis spectra should be performed to eliminate the errors and particle effect and (3) forecast methods should be developed to employ online monitoring in real-time for water quality monitoring and water treatment process control. There are two approaches to applying the online UV-Vis instruments for water quality measurements: direct water quality parameter measurements from the built-in algorithms and indirect measurements through the combinations of chemometrics and the UV-Vis absorbance time series from the instruments.

### 4.3. Future Research of Online UV-Vis Spectrophotometers

The future trend of the application of online UV-Vis instruments is that the instrument will be a key component for water quality monitoring as a part of drinking water quality management. As most of the reported studies on the use of online UV-Vis instruments were conducted at a laboratory- or pilot-scale, future work is needed, particularly for large-scale applications, such as field applications. To have correct measurements, it is necessary to have trials when the instruments are used for new sites. A site-specific particle compensation (calibration) may be needed. The differences in the methods of determination of various water quality parameters is also a challenge for practical applications. Further studies are necessary to find out the best solutions for the specific applications. A possible solution is the detection of water quality parameters based on multiple data fusion technology. It evaluates the analysis of different water quality parameters data and extracts more completed information than a single data source [8].

Future research needs to include the progress in the field application of UV-Vis instruments. Real-time monitoring using a UV-Vis instrument combined with advanced data processing can provide real-time measurements for rapid data analysis, which, in turn, contributes to the real-time water quality management system. Integration of the instrument and data analytics for data pre-treatment and processing is a key factor for measuring UV-Vis spectra in real-time [8], allowing anomaly detection and building early warning systems. Data analytics of water quality data using the UV-Vis instruments combined with data platforms have capabilities to automatically analyse and correct data in real-time and then predict to improve water quality monitoring and process control. Since each water quality detection method based on the UV-Vis spectra has its strengths and drawbacks, multiple methods should be conducted to assess their performance and analyse which methods can be used to construct decision support tools for the optimisation of a particular WTP [81]. The future application of the UV-Vis instrument also includes exploring the use of raw spectra as inputs to determine the measurements of other water quality parameters. With the assistance of chemometrics such as PLS and artificial neural networks [61], more accurate measurements can be obtained [9].

## 5. Conclusions

This review covers the practical aspects of the employment of online UV-Vis spectrophotometers for water quality monitoring and process control, particularly techniques for industrial applications. The use of online UV-Vis spectrophotometers for drinking water quality management in the literature has been discussed. Commonly employed online UV-Vis instruments for drinking water have been discussed. Water quality parameters, including UV_254_, colour, DOC, turbidity and nitrate, can be directly generated from the built-in algorithms of the online UV-Vis instruments. Site-specific calibrations can be conducted to improve the accuracies of the measurements if the generic built-in algorithms are under-performing for a water source. Alternative particle compensation methods to the built-in particle compensation method have been discussed. These methods are based on the UV-Vis spectra of water and chemometrics, which offer simplicity and flexibility of removing the particle effect from the measurements. Various techniques of anomaly detection and early warning were also discussed to monitor water quality at the source or in the distribution system for water quality control as a part of the drinking water quality management system. As most studies of online UV-Vis instruments in the drinking water area were at the laboratory- and pilot-scale, future work is needed, particularly for industrial-scale applications. Issues and potential solutions of using the online instruments were provided. Future research also needs to work towards the integration of early warning and real-time water process control systems for water quality management.

## Figures and Tables

**Table 2:** Summary of indirect particle compensation methods for online water quality monitoring.

Methods	Wavelengths (nm)	Parameter	Data Type	Sources	Literature
SW	350 nm	COD	Lab	Ground water	[47]
SW	546 nm	COD	Lab	Simulated water samples	[48]
SW	545 nm	UV_254_	Lab and field	Surface water	[49]
SW, MSC	550 nm	UV_254_	Field	Surface water, treated water	[27]
Two wavelengths	254, 340 nm	DOC	Field data	Surface water	[50]
MSC	Full spectra	COD	Lab	Stream water, Simulated water	[51]
PLS	200–400 nm	COD	Lab	Lake water	[19]
PLS	Full spectra	DOC		River water	[52]
PLS	full	Nitrate	Lab	Simulated water	[53]
PLS	380–750 nm	Nitrate, TOC, COD	Lab	Seawater	[54]
PLS	Full spectra	assimilable organic carbon	Pilot	Simulated lake water	[55]
MSC, PLS, PCR	250–740 nm	DOC	Field	Surface water	[18]
PLS, lasso regression and MSR	Full spectra	Nitrate, DOC	Field	Brackish water	[56]
MSR	250, 290, 307.5, 437.5, 447.5, 630, 645 nm	DOC, Fe	Lab and Field	Stream water	[17]
PLS, MSR, local and global	250–740 nm	DOC	Field	Surface water	[57]
Multiple linear regression	260, 265, 280 and 285 nm	TOC	Lab	Drinking water, seawater, river water	[21]
SVM	Full spectra	Nitrate	Lab	River water	[58]

**Table 4:** Challenges and solutions of using the online UV-Vis spectrophotometers.

Challenges	Causes	Solutions	Source
Installation issues	Probe corrosion issue	Use an ‘on-demand’ pump sampling system	[38]
Measurement accuracy	Missing calibrations Low water level	Proper calibration in-situ and maintenance Pump water to the instrument	[93]
Detection difficulty	Challenging nature of the source water	Site-specific compensation Regular maintenance Select the correct pathlength Develop surrogate parameters	[41,82,90]
Data processing	Large volume of data Data type Faulty data	Use or develop specialised tools Expertise	[3,38,78,90]
Maintenance cost	Calibration issue	Use alternative particle compensation method Provide training for maintenance skills	[38]
